# Magnetic resonance imaging evaluation of the distal oblique bundle in the distal interosseous membrane of the forearm

**DOI:** 10.1186/s12891-017-1419-2

**Published:** 2017-01-26

**Authors:** Yeon Ho Kim, Hyun Sik Gong, Jin Woo Park, Hyun Kyung Yang, Kahyun Kim, Goo Hyun Baek

**Affiliations:** 1 Department of Orthopedic Surgery, Seoul National University Bundang Hospital, Seongnam, South Korea; 2 Department of Radiology, Seoul National University Bundang Hospital, Seongnam, South Korea; 3 Department of Orthopedic Surgery, Seoul National University Hospital, Seoul, South Korea; 4 Department of Orthopedic Surgery, Seoul National University Bundang Hospital, Seoul National University College of Medicine, 300 Gumi-dong, Bundang-gu, Seongnam-si, Gyeonggi-do 463-707 South Korea

**Keywords:** Distal interosseous membrane, Distal oblique bundle, Distal radioulnar joint, Magnetic resonance imaging

## Abstract

**Background:**

Some cadaveric studies have reported the role of the distal oblique bundle (DOB) in the distal radioulnar joint stability. We aimed to determine whether the presence of the DOB can be identified and its thickness can be measured in magnetic resonance imaging (MRI) examinations.

**Methods:**

We retrospectively reviewed 468 wrist and forearm MRIs. Inclusion criteria were wrist or forearm MRIs taken in patients older than 18 years of age, and exclusion criteria were patients with acute wrist or forearm fractures, infections, or malignant tumors. We selected 80 MRIs that provided adequate coverage of the distal interosseous membrane (DIOM). The thickness of the DIOM in the T2-weighted transverse plane was measured on the picture archiving and communicating system. We used a model-based clustering method to determine whether some individuals have thicker DIOMs that can be considered as the DOB.

**Results:**

The thickness of the DIOM demonstrated a bimodal distribution, indicating the presence of patients with a thick DIOM (DOB). The model-based clustering method indicated that the optimal cutoff point was 1.0 mm. Twenty-six individuals (32.5%) had thick DIOMs with a mean thickness of 1.4 mm (standard deviation, 0.2 mm), while 54 individuals (67.5%) had thin DIOMs with a mean thickness of 0.6 mm (standard deviation, 0.2 mm).

**Conclusion:**

Our study demonstrates that it is possible to identify the DOB and measure its thickness using MRI. Future in-vivo studies of the DOB using MRI in patients with distal radioulnar joint pathologies may reveal its role in the distal radioulnar joint stability.

## Background

The interosseous membrane of the forearm is a ligamentous complex connecting the radius to the ulna, and it consists of distal membranous, middle ligamentous, and proximal membranous portions. The distal interosseous membrane (DIOM) acts as a secondary stabilizer of the distal radioulnar joint (DRUJ) when the dorsal and palmar radioulnar ligaments of the triangular fibrocartilage complex (TFCC) are cut [1, 2]. Noda et al. reported that the thickness of the DIOM varied widely among the specimens and identified the distal oblique bundle (DOB), which is a thick fiber running within the DIOM that originates from the distal one-sixth of the ulnar shaft and runs distally to insert on the inferior rim of the sigmoid notch of the radius [3]. A biomechanical study found that the DOB had little changes in length during forearm rotation, suggesting that it is an isometric stabilizer of the forearm [4].

Recently, studies have reported the role of the DOB in the DRUJ stability. Kitamura et al. reported in a cadaveric study that DRUJ laxity was greater in the group without a DOB than in the group with a DOB [5]. Arimitsu et al. found in another cadaveric study that ulnar shortening with the osteotomy carried out proximal to the attachment of the DIOM had a more favorable effect on DRUJ stability compared with distal osteotomy, especially when there was a DOB [6]. In addition, Dy et al. demonstrated that in the setting of an ulnar styloid fracture, coronal shift of the distal radius fracture is associated with increased DRUJ instability in specimens with a distinct DOB, but not in specimens without a distinct DOB [7]. Furthermore, Moritomo suggests that a thick DIOM (or DOB) has an important role in stabilizing the ulnar stump after the Sauvé-Kapandji procedure [8]. However, these studies are done in cadavers, and it is still unknown whether the presence or absence of the DOB affects clinical outcomes. Therefore, identifying the DOB clinically would be important for future clinical studies exploring the role of this structure in DRUJ stability.

Several studies reported that magnetic resonance imaging (MRI) could evaluate an intact or disrupted interosseous membrane in its central or proximal part in the setting of longitudinal forearm instability (Essex-Lopresti lesion) [9–11]. We hypothesized that MRI might also evaluate the interosseous membrane at its distal part. The purpose of this study was to determine whether the presence of the DOB can be identified and its thickness can be measured in MRI examinations.

## Methods

### Subjects

We obtained an approval for this study from our institutional review board. Using the electronic medical data record program, we retrieved the list of all patients who had undergone MRI examinations between March 2003 and March 2015 at our hospital, which is an urban tertiary referral hospital. Inclusion criteria were wrist or forearm MRIs taken in patients older than 18 years of age, and exclusion criteria were patients with acute wrist or forearm fractures, infections, or malignant tumors. Thus, 468 wrist and forearm MRIs were retrieved from the data and reviewed.

In these 468 MRIs, we examined whether the MRI provided sufficient coverage of the length of the DIOM that originates from the distal one-sixth of the ulnar shaft and runs toward dorsal and distal direction to insert on the dorsal inferior rim of the sigmoid notch of the radius. A previous anatomical study reported that the mean proximal edge of the DIOM was 59 mm (range, 53–63 mm) proximal to the ulnar head [5]. Therefore, we selected MRIs that covered the forearm from the wrist to at least 60 mm proximal to the ulnar head. Finally, 80 MRIs (17%) that provided adequate resolution and coverage of the DIOM were included in the study. The mean age of the patients was 46. 9 years (range, 22–86 years). There were 38 men (47.5%) and 42 women (52.5%). The diagnoses of the patients included benign soft tissue mass in 38 patients (47.5%), TFCC lesions in 16 patients (20%), peripheral neuropathy in 7 patients (8.8%), Kienböck disease in 5 patients (6.3%), scaphoid nonunion in 4 patients (5%), and other conditions with wrist pain in 10 patients (13%).

### MRI protocol and measurement

**Fig. 1:**
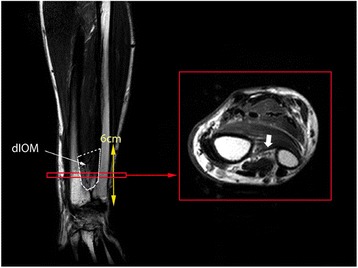
The distal interosseous membrane (DIOM), which is schematically represented in the coronal image of the forearm (*white dotted area*), was traced in the transverse images of the forearm from proximally to distally within the range of the DIOM (*yellow arrow*). The thickest part of the DIOM was chosen from the transverse images and the thickness was measured (*white arrow*)

All MR examinations were performed on a 3.0T unit (Achieva & Ingenia; Philips Healthcare, Best, the Netherlands) with the subject in the supine position. Images were taken according to the standard MR protocol for wrist and forearm imaging in our hospital. The DIOM was identified on T2-weighted Turbo Spin-echo (TSE) imaging in a transverse plane (2–6 mm thickness sections; repetition time (TR) / echo time (TE), 2694-5011/80-100 msec; field of view of 80-150 × 80-150 or 160-320 × 160-320 matrix). The DIOM was seen as a linear low signal intensity band between the radius and the ulna. As the DOB runs obliquely from the ulna proximally to the radius distally, no single transverse cut can show the thick part in the entire length of the DIOM. An anatomic study reports that the DOB showed variations; the DOB can be linear, can spread in a fan shape distally, or can occupy the whole DIOM [5]. Therefore, we traced the thick part from proximal, ulnar side to the distal, radial side, and measured the maximum thickness by using the picture archiving and communicating system in every transverse cut from proximally (6cm proximal to the ulnar styloid) to distally (sigmoid notch of the radius) within the range of the DIOM. The data of the thickest part of the DIOM were collected and analyzed (Fig. 1).

Two authors (one orthopedic surgeon and one radiologist), who were blinded to patient information, measured the DIOM thickness. We evaluated the intra-rater reliability by repeating all measurements after 2 weeks, and inter-rater reliability by the independent assessment by 2 examiners. The intra- and inter-rater reliabilities of the thickness measurement were tested using intraclass correlation coefficients (ICCs).

### Statistical analysis

Model-based clustering method (Mclust package in R) was used to determine the optimal number of Gaussian mixtures and the cutoff value based on the Bayesian information criterion (BIC). This statistical method demonstrates the number of clusters in the distribution [12], and is often used for medical statistical analysis [13, 14]. We compared the thickness of the DIOM between groups using the *t*-test, and frequency of the DOB between groups using the chi-square test. A power analysis indicated that a sample size of 18 patients to each of the group would provide 90% statistical power (α = 0.05; ß = 0.10) with use of *t*-test for an effect size of 1.0 (0.2mm difference between the groups with a standard deviation of 0.2mm).

We used two types of MRI scanners (Achieva and Ingenia), and the section thickness varied from 2 to 6mm. Therefore, we compared the DOB thickness between the two scanners and between sections of 3mm or thinner and sections of more than 3mm.

## Results

### Reliability of measurements

The ICC value of intra-rater reliability was 0.833, and that of inter-rater reliability was 0.748. As these ICC values indicated high reliability, we used the thickness measured by one of the authors.

### Identification of the DOB

**Fig. 2:**
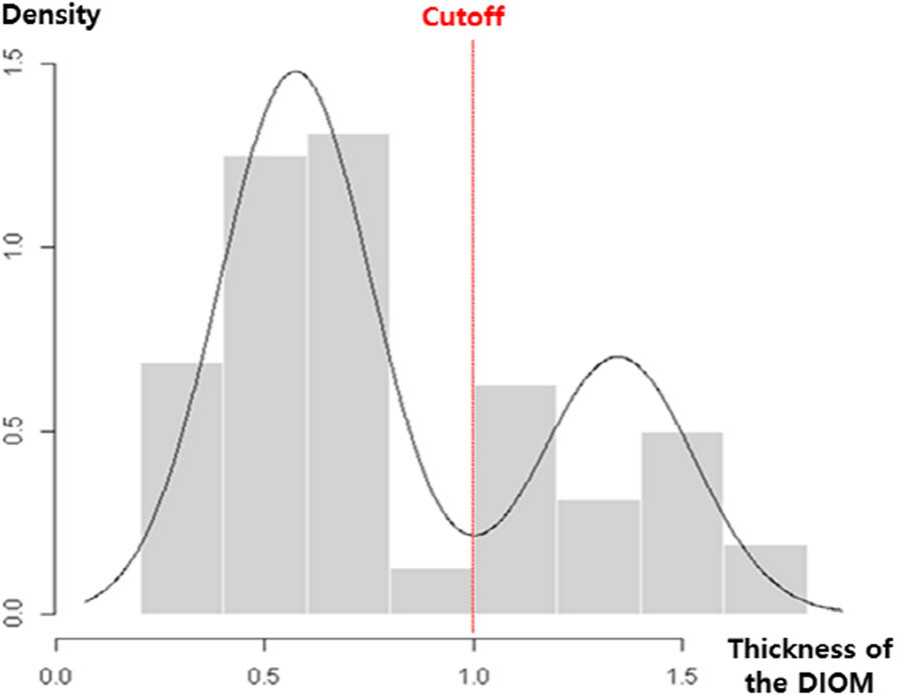
The density plot of the thickness of the distal interosseous membrane (DIOM) and the cutoff value based on the Model-based clustering method. The optimal cutoff point was suggested as 1.0 mm

The thickness of the DIOM demonstrated a bimodal distribution, indicating the presence of two groups that had a normal distribution. The two groups were divided on the basis of a cutoff point which was established by the model-based clustering method and the optimal cutoff point was suggested as 1.0 mm (Fig. 2). The group with a thick DIOM was considered to have the DOB.

### Frequency and thickness of the DOB

**Fig. 3:**
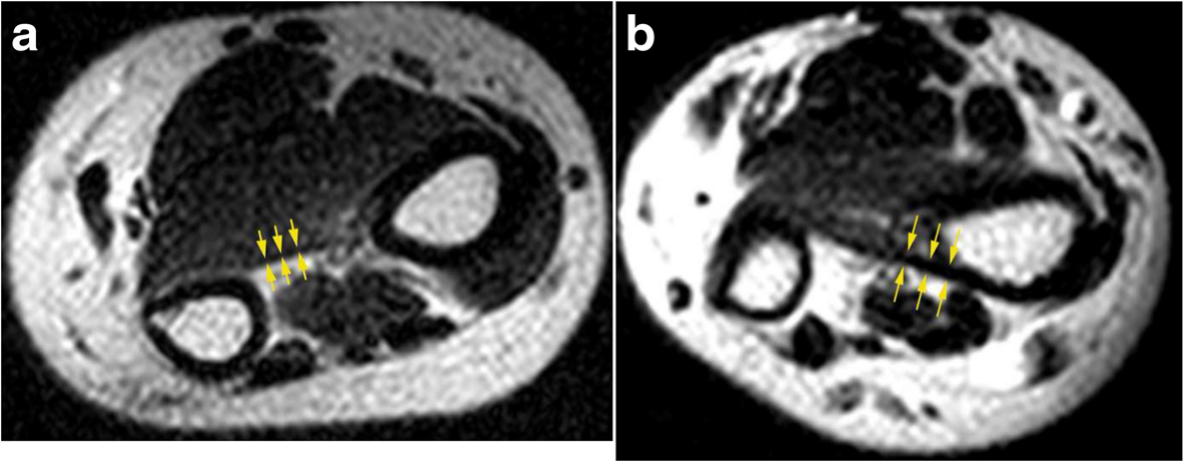
Two representative cases of the patients with a thin DIOM (**a**, no-DOB Group, 54-year-old female) and with a thick DIOM (**b**, DOB Group, 25-year-old female). The DIOMs were measured 0.67 mm thick (**a**) and 1.32 mm thick (**b**) respectively

The number of patients who had a thicker DIOM (DOB Group) was 26 (32.5%) and that of patients who had a thinner DIOM (no-DOB Group) was 54 (67.5%). The mean thickness of the DIOMs in the DOB group was 1.4 mm (standard deviation, 0.2 mm; range, 1.1–1.7 mm), and that in the no-DOB group was 0.6 mm (standard deviation, 0.2 mm; range, 0.2–0.9 mm) (Fig. 3). The mean thickness was significantly different between the two groups (*p* = 0.044).

There were no significant differences in the frequency of the DOB (*p* = 0.549), or in the thickness of the DIOM (*p* = 0.716) between men and women. In addition, there were no significant differences in the frequency and thickness according to the scanner type (Achieva and Ingenia) or section thickness (equal to or less than 3mm vs. more than 3mm) (Table 1).

**Table 1:** Comparison according to scanner type, sex, and section thickness

		Mean Thickness of the DIOM	Frequency of the DOB
Scanner type	Achieva (*n* = 32)	0.89mm	15
	Ingenia (*n* = 48)	0.77mm	11
*P* value		0.772	0.604
Sex	Men (*n* = 38)	0.80mm	13
	Women (*n* = 42)	0.84mm	13
*P* value		0.716	0.549
Section thickness	>3mm (*n* = 51)	0.86 mm	18
	≤3mm (*n* = 29)	0.74 mm	8
*P* value		0.157	0.479

## Discussion

Cadaveric studies have reported the presence of the DOB within the DIOM and its potential role in stabilizing the DRUJ. Identification of the DOB by an imaging modality in clinical studies may further clarify its role in the DRUJ stability, and may allow better prediction of the prognosis or planning of the surgery. Our study demonstrates that the presence of the DOB can be identified and its thickness can be measured in the T2-weighted transverse plane of MRI that provides adequate coverage of the wrist and the distal forearm.

In our study, the frequency of the DOB was 32.5% (26 out of the 80 patients), which is comparable to previous findings in cadaveric studies [3, 5]. Noda et al. reported an frequency of 40% (12/30) in a study using 30 forearms from 15 embalmed cadavers (9 females and 6 males, mean age 85 years), [3] and Kitamura et al. found that 4 out of the 10 fresh-frozen cadavers (5 females and 5 males, mean age 79 years) had the DOB [5]. Our study found that the mean thickness of the DIOM in the DOB group was 1.4 mm and that in the no-DOB group was 0.6 mm, which were comparable to the study by Kitamura et al, where these values were 1.2 mm and 0.4 mm, respectively [5]. The mean thickness of the DOB was 1.5 mm in the study by Noda et al. [3].

Studies suggest that identifying the presence or absence of the DOB by MRI preoperatively may be helpful in decision-making for patients with ulnar-sided wrist pain or DRUJ problems. Arimitsu et al. found that the DRUJ stability is improved when ulnar shortening osteotomy is performed proximal to the ulnar attachment of the DIOM [6]. They suggest that an enhanced DRUJ stabilizing effect can be expected in patients having a DOB, and an additional or alternative procedure may be considered when the DRUJ instability remains a concern in patients without a DOB [6]. On the other hand, they found significant differences in the longitudinal closure difficulty for proximal compared with distal osteotomy sites, depending on the DOB thickness. Accordingly, they suggest that for patients with a stable DRUJ, an osteotomy performed distal to the ulnar attachment of the DOB may allow better healing of the osteotomy site. A few studies report the advantages of distal metaphyseal ulnar shortening osteotomy [15, 16]. However, the study by Arimitsu is a cadaveric study based on 10 cases. Future clinical studies might determine the clinical relevance of the DOB in ulnar shortening osteotomy, by identifying the presence of the DOB and correlating the outcomes.

The DIOM originates from the distal ulna on average 59 mm (range, 53–63 mm) proximal to the ulnar head [5]. We selected MRIs that covered the forearm from the wrist to at least 60 mm proximal to the ulnar head, and it was possible to see the DIOM in all of the selected MRIs. Previous studies on the IOM also demonstrated that IOM injury was detected using the T2-weighted sequence with a reduced slice thickness [10, 11]. In addition, Fast Spin-echo (FSE) technique, which is similar to the TSE technique used in our study, was reported to yield clearer images by limiting distortion and to allow the images to be produced more quickly [9]. Our study suggests that changing the MRI protocol to include at least 60 mm proximal to the ulnar head and T2-weighted TSE (or FSE) imaging may be necessary to check the status of the DOB in patients presenting with DRUJ problems. Okada et al. evaluated the DIOM in 14 patients using ultrasound, and the sensitivity of ultrasound evaluation in confirming presence of the DOB was 80% [17]. Although ultrasound can be a useful and relatively inexpensive tool in detecting the presence of DOB, MRI can evaluate most of the cartilage and ligament lesions and does not depend on the competence of examiners.

There are a few limitations to this study. First, we evaluated patients who underwent the MRI examination for wrist or forearm problems. The prevalence of the DOB in the general population needs to be investigated further. Furthermore, Most of the wrist MRIs were excluded because they did not cover the distal forearm proximally long enough, which could be a selection bias. Second, our study identified the DOB on imaging only. Studies using both the MRI and surgical findings should confirm the accuracy of the MRI findings. However, evaluation of the DIOM involves elevation of the deep forearm muscles, thus it could be difficult to examine the DIOM for study purposes in patients with common wrist conditions. Third, we collected cases from 12 years period and therefore there must have been lots of variability in the MRI pulse sequence parameters. Forth, although the thickness of the DIOM and the frequency of the DOB were not different between sections of thin and thick sections, thin sections less than 3mm would be ideal for accurate measurement of the DIOM thickness. Finally, we could not stadiardise the position of the forearm in relation to supination/pronation, which may have affected the thickness measurement. However, the DOB was shown to be isometric during forearm rotation, [4] and we did not observe any wavy deformity of the DOB in the transverse planes.

## Conclusions

This study demonstrates that it is possible to identify the DOB and measure its thickness using MRI. The frequency and thickness of the DOB in our study support the findings of previous cadaveric studies on its morphology. Future in-vivo studies of the DOB using MRI in patients with distal radioulnar joint pathologies may reveal its role in the distal radioulnar joint stability.

## Abbreviations

DIOM: Distal interosseous membrane
DOB: distal oblique bundle
DRUJ: distal radioulnar joint
ICCs: intraclass correlation coefficients
